# Evaluation of Bubble Entropy Using Heart Rate Variability

**DOI:** 10.3390/e28060638

**Published:** 2026-06-05

**Authors:** Dimitrios Platakis, Roberto Sassi, George Manis

**Affiliations:** 1 Department of Computer Science and Engineering, University of Ioannina, 45500 Ioannina, Greece; d.platakis@uoi.gr; 2 Department of Computer Science, University of Milan, 20133 Milano, Italy; roberto.sassi@unimi.it

**Keywords:** entropy, bubble entropy, sample entropy, approximate entropy, permutation entropy

## Abstract

Bubble entropy has established its own place in the research community, representing a new and promising definition of entropy. Based on the work required to order a vector in an embedding space of dimension *m*, Bubble entropy gives a physical interpretation of what the metric actually computes. In this work, Bubble entropy is evaluated based on its ability to classify RR time series, the time series most commonly considered for entropy-based analysis in the field of biomedical engineering. For this purpose, it is compared with three other definitions of entropy: the most widely used Sample entropy and Approximate entropy, the most relative to Bubble entropy, and also the widely used Permutation entropy. Signals from healthy individuals, in sinus rhythm, are compared with signals from cardiac patients, and machine learning methods are applied to calculate the classification accuracy that each method can achieve. The classifiers chosen are k-Nearest Neighbors, Support Vector Machine, Logistic Regression, and Gaussian Naive Bayes. Feature evaluation methods are also employed to serve as additional measures of effectiveness. Bubble entropy generally manages to achieve better results than Sample entropy, Approximate entropy and Permutation entropy, both in terms of classification accuracy and feature ranking.

## 1. Introduction

Bubble entropy (*BubbleEn*) [1,2,3] was introduced in 2017 [1] as an entropy measure “almost free of parameters.” It was specifically designed to relieve researchers of the need for parameter estimation, which is often a source of bias and faulty estimations in traditional entropy measures. Unlike conventional approaches that compute entropy, based on the distribution of values or sorting patterns, Bubble entropy evaluates the distribution of the computational effort required to sort the elements of each vector in the embedding space.

The key idea is that the “task”—defined as the number of swaps performed by the bubble sort algorithm—corresponds to the energy expenditure required to reorder the vector. This provides a physical interpretation: rather than abstractly counting patterns, Bubble entropy quantifies the “work” needed to impose order, which is closely linked to entropy in physical systems. Using swaps as a measure of effort also reduces the number of possible states in the resulting distribution, producing a probability distribution compared to the large number of patterns appearing in methods like Permutation entropy [4]. The probability mass function of the observed swaps is then used to compute the second-order Rényi entropy. Bubble entropy is defined as the normalized difference between the entropy in spaces with dimension m+1 and *m*.

Since its emergence, Bubble entropy has found applications across many research fields, including biomedical engineering. In particular, it has been used across a range of physiological applications. Electroencephalograms have been examined during sleep [5,6], for mild cognitive impairment [7] or vascular dementia assessment [8] and speech recognition [9]. Heart rate variability signals obtained from ECG signals [10,11,12] and cardiotocograms [13] have also been discriminated. Furthermore, Bubble entropy has been used in PPG signals [14] and in electrohysterography [15,16]. It should be noted that it has served as the foundation for numerous novel methods that build upon its core concept, which have extended beyond the field of biomedical engineering, including [17,18,19].

In this paper, we assess the ability of Bubble entropy to discriminate RR time series (time intervals between successive heartbeats). Recordings from healthy individuals serve as controls. Patients with congestive heart failure and atrial fibrillation, or patients who experienced sudden cardiac death, are the pathological classes. Features extracted using Sample entropy [20,21], Approximate entropy [22], Permutation entropy [4], and Bubble entropy are evaluated and selected before being fed as input to several classifiers. The evaluation is based on the computed accuracy, weighted F1-score and feature selection processes. Comparative results are presented and discussed, before conclusions are drawn. Bubble entropy was shown to be a promising method for the analysis of RR time series and, in most cases, it outperforms the three other examined methods.

The contribution of this paper focuses on the evaluation of Bubble entropy and can be summarized as follows:

- In [3], a limited comparison with Sample entropy and Permutation entropy was performed, based only on one classification problem with one pathological dataset. In this paper, six classification challenges are examined and an additional evaluation metric has been employed, to increase the reliability of the comparisons.
- In this paper, Approximate entropy is added to the examined methods, completing the set of the most common, widely used and well-established entropy definitions.
- In addition, four ranking measures were applied for the evaluation of the extracted features, increasing the credibility of the experiments.
- While in [3] feature evaluation was limited, in this paper the features are assessed independently, based on their capability to perform accurate classification, solely, without the contribution of other entropy features.

The need for a more detailed assessment of Bubble entropy becomes evident from the method’s acceptance by the research community. Given the number of research works having adopted the method and the number of modifications or extensions appearing in the literature [23], a broader analysis of its most common application in biomedical engineering, namely, heart rate variability, emerges.

In the rest of the paper, we first provide the definitions of the four methods under comparison (Section 2), followed by a description of the methodology and datasets employed (Section 3). The results of the study are then presented (Section 4) and discussed (Section 5), before the last section (Section 6) concludes with a summary of the main findings.

## 2. Definitions

In this section, short descriptions of Sample entropy, Approximate entropy, Permutation entropy and Bubble entropy will be given.

### 2.1. Sample Entropy

Let $\left\{x_{1},x_{2},\ldots ,x_{N}\right\}\in R^{N}$ be a scalar time series. The *m*-dimensional embedding vectors are defined as follows:

$$u\left(i\right)=\left[x_{i},x_{i+1},\ldots ,x_{i+m-1}\right],\;1\leq i\leq N-m+1.$$

Distances are measured using the infinity norm of the difference between two vectors:

$$d\left(u\left(i\right),u\left(j\right)\right)=\left||u\left(i\right),u\left(j\right)|\right|=\max_{0\leq k\leq m-1}\left|x_{i+k}-x_{j+k}\right|.$$

Two vectors u(i),u(j) are similar when

||u(i),u(j)||≤r.

We count similar vectors in dimension *m*:

$$B_{i}^{\left(m\right)}\left(r\right)=\frac{1}{N-m}\sum_{\begin{array}{c} j=1\\ j\neq i \end{array}}^{N-m}1\;\left[d(u(i),u(j))\leq r\right],$$

where 1[·] is equal to 1 when the condition in square brackets holds, or 0 otherwise. Next, we compute the following:

$$B^{\left(m\right)}\left(r\right)=\frac{1}{N-m}\sum_{i=1}^{N-m}B_{i}^{\left(m\right)}\left(r\right).$$

Similarly for dimension m+1,

$$A_{i}^{\left(m\right)}\left(r\right)=\frac{1}{N-m}\sum_{\begin{array}{c} j=1\\ j\neq i \end{array}}^{N-m}1\;\left[d(v(i),v(j))\leq r\right],$$

where v are vectors in m+1 dimensional space, and

$$A^{\left(m\right)}\left(r\right)=\frac{1}{N-m}\sum_{i=1}^{N-m}A_{i}^{\left(m\right)}\left(r\right).$$

Sample entropy is reported as follows:

$$\mathrm{SampEn}\left(m,r,N\right)=-\ln\;\left(\frac{A^{\left(m\right)}\left(r\right)}{B^{\left(m\right)}\left(r\right)}\right).$$

### 2.2. Approximate Entropy

We use the same definitions with Section 2.1. For each vector u(i), the fraction of vectors u(j) (including j=i) that are similar is as follows:

$$C_{i}^{\left(m\right)}\left(r\right)=\frac{1}{N-m+1}\sum_{j=1}^{N-m+1}1\;\left[d(u(i),u(j))\leq r\right].$$

We define

$$\Phi^{\left(m\right)}\left(r\right)=\frac{1}{N-m+1}\sum_{i=1}^{N-m+1}\ln C_{i}^{\left(m\right)}\left(r\right).$$

Approximate entropy is then given by the following:

$$\mathrm{ApEn}\left(m,r,N\right)=\Phi^{\left(m\right)}\left(r\right)-\Phi^{\left(m+1\right)}\left(r\right),$$

where $\Phi^{\left(m+1\right)}\left(r\right)$ is computed in the same way but for embedding dimension m+1.

### 2.3. Permutation Entropy

We also use the same definitions here. For each time index *i* with 1≤i≤N−m+1, define the embedding vector:

$$u\left(i\right)=\left[x_{i},\;x_{i+1},\;x_{i+2},\;\ldots ,\;x_{i+m-1}\right].$$

Each vector u(i) induces a permutation π(i) of {1,2,…,m} determined by the rank order of its components:

$$x_{i+(k_{1}-1)}\leq x_{i+(k_{2}-1)}\leq \cdots \leq x_{i+(k_{m}-1)}\;\Rightarrow \;\pi \left(i\right)=\left(k_{1},k_{2},\ldots ,k_{m}\right).$$

Let $S_{m}$ denote the set of all m! permutations of length *m*. For each $\pi \in S_{m}$, define its empirical probability:

$$p\left(\pi \right)=\frac{1}{N-m+1}\sum_{i=1}^{N-m+1}1\;\left[\;\pi (i)=\pi \;\right].$$

The permutation entropy is the Shannon entropy of this distribution:

$$PermEn\left(m\right)=-\sum_{\pi \in S_{m}}p\left(\pi \right)\;\ln p\left(\pi \right).$$

### 2.4. Bubble Entropy

Let $\left\{x_{1},x_{2},\ldots ,x_{N}\right\}\in R^{N}$ be a scalar time series. Fix an embedding dimension m∈N. For each time index *i* with 1≤i≤N−m+1, define the embedding vector:

$$u\left(i\right)=\left[x_{i},\;x_{i+1},\;x_{i+2},\;\ldots ,\;x_{i+m-1}\right].$$

For each u(i), let b(i) denote the number of adjacent transpositions (bubble sort swaps) required to sort u(i) into nondecreasing order.

Let $K_{\max}=\frac{m(m-1)}{2}$ be the maximum possible inversion count. For each $k\in \{0,1,\ldots ,K_{\max}\}$ define the empirical probability:

$$p\left(k\right)=\frac{1}{N-m+1}\sum_{i=1}^{N-m+1}1\;\left[\;b(i)=k\;\right].$$

The Swap entropy is the Rényi entropy of this distribution:

$$H_{\mathrm{swap}}\left(m\right)=-\log\sum_{k=0}^{K_{\max}}p{\left(k\right)}^{2}.$$

Bubble entropy is reported as the difference in entropy in spaces with sizes *m* and m+1:

$$BubbEn\left(m\right)=H_{\mathrm{swap}}\left(m+1\right)-H_{\mathrm{swap}}\left(m\right).$$

In [2], three normalization methods are examined for Bubble entropy. Normalization methods do not affect the discriminative power of the method, only the reported values. In this paper, we chose to use the following one:

$$BubbEn\left(m\right)=\left(H_{\mathrm{swap}}\left(m+1\right)-H_{\mathrm{swap}}\left(m\right)\right)/\log\frac{m+1}{m-1}.$$

## 3. Data and Methods

### 3.1. Datasets

For our evaluation, we employed four datasets with ECG (RR) time series, publicly available on PhysioNet [24]. Each dataset includes recordings from a different group of subjects, as described in the following.

The first dataset comprises 72 long-term (24 h approximately) heartbeat time series. It was created by combining two smaller datasets, referred to as “NSRDB” (Normal Sinus Rhythm RR Interval Database) [24] and “NSR2DB” (Normal Sinus Rhythm RR Interval Database 2) [24] on PhysioNet. “NSRDB” includes 18 long-term ECG recordings, 5 men, aged 26 to 45, and 13 women, aged 20 to 50. “NSR2DB” includes beat annotation files for 54 long-term ECG recordings of subjects in normal sinus rhythm, 30 men, aged 28.5 to 76, and 24 women, aged 58 to 73. We will refer to the whole dataset, with the 72 recordings, as the “NSR” dataset and consider it as our control group.

The second dataset consists of 44 long-term heartbeat (24 h approximately) from patients with congestive heart failure. It combines two datasets available on Physionet, referred to as “CHFDB” (Congestive Heart Failure Database) [24,25] and “CHF2DB” (Congestive Heart Failure) [24]. “CHFDB” includes 15 subjects, 11 men, aged 22 to 71, and 4 women, aged 54 to 63 with severe congestive heart failure (NYHA class 3–4). “CHF2DB” includes heartbeat time series for 29 long-term ECG recordings of subjects aged 34 to 79, with congestive heart failure (NYHA classes I, II, and III). Subjects included 8 men and 2 women; gender is not known for the remaining 19 subjects. We will refer to the whole dataset, with the 44 recordings, as the “CHF” dataset and consider it as one of our patient groups.

The third dataset is the MIT-BIH Atrial Fibrillation Database. We will refer to it as “AF” [24,26]. It comprises 25 long-term ECG recordings of human subjects with atrial fibrillation (mostly paroxysmal). The recordings are each 10 h in duration. The rhythm annotation files were prepared manually. They contain rhythm annotations of types AFIB (atrial fibrillation), AFL (atrial flutter), J (AV junctional rhythm), and N, which indicate all other rhythms. Beat annotation files were prepared using an automated detector and have not been corrected manually.

The fourth dataset is the Sudden Cardiac Death Holter Database [24,27]. It is a database with 23 complete Holter recordings. The database currently includes 18 patients with underlying sinus rhythm (4 with intermittent pacing), 1 who was continuously paced, and 4 with atrial fibrillation. All patients had a sustained ventricular tachyarrhythmia, and most had an actual cardiac arrest. We will refer to this database as “SCD”.

In this study, all ECG signals were transformed into RR interval series and Table 1 describes the key statistical properties of the databases. Mean values (measured in seconds) and standard deviations are given for completeness, while the mean and the minimum signal length in each dataset are also available.

### 3.2. Methodology

Our evaluation strategy is based on assessing the features extracted by each method. The extent to which each method can achieve high-accuracy classification using only the features it generated is the main criterion. The comparison between each method’s standalone classification accuracy and the accuracy obtained when all methods are combined is another evaluation criterion.

At the feature level, all extracted features are assessed using standard statistical measures. The statistical relevance and predictive association of features, as well as its capability to contribute to a machine-learning-based classification, constitute an additional evaluation criterion.

A critical issue is the selection of the parameters that will be used for each method. For Permutation entropy and Bubble entropy, the problem is easy and clear: all potentially informative values of *m* will be examined. Since *m* is an integer number, this is not a problem. However, for Sample entropy and Approximate entropy, the threshold distance *r* is involved, which is a real number, something that makes it impossible to probe all potentially informative values. The most straightforward choice was to select values based on a typical value (r=0.2) and values around it. For the parameter *m*, the problem was again easy, since it was possible for all potentially informative values of *m* to be included.

Thus, for Sample entropy, 16 features were computed, with all combinations of *m* = 1, 2, 3, 4 and *r* = 0.15, 0.20, 0.25, 0.30 being examined. The same 16 features were computed for Approximate entropy (*m* = 1, 2, 3, 4 and *r* = 0.15, 0.20, 0.25, 0.30). The first 15 values of *m* (*m* = 2…16) were enough for Permutation entropy, since for larger values of *m*, it is unfeasible to get proper estimates, as noted earlier. Finally, the first 30 values of *m* (*m* = 2…31) were included for Bubble entropy, given that the values around m=20 can still provide useful information. The reason for extending *m* to 31 is twofold. First, we aimed to maintain a sufficiently wide margin beyond the “useful” embedding dimensions, given that if no informative features appeared there, feature selection mechanisms would naturally disregard them. Second, as noted in the paper, the computation of Bubble entropy features is inexpensive, so there is no practical drawback to including them. A similar wide margin was used for Permutation entropy, where embedding dimensions m<10 proved usually effective in previous studies.

We used the datasets described in Section 3.1. We created three two-class classification challenges and three three-class classification challenges, each comparing the NSR dataset, considered as the control group, with other datasets or combinations of them.

**Figure 1:**
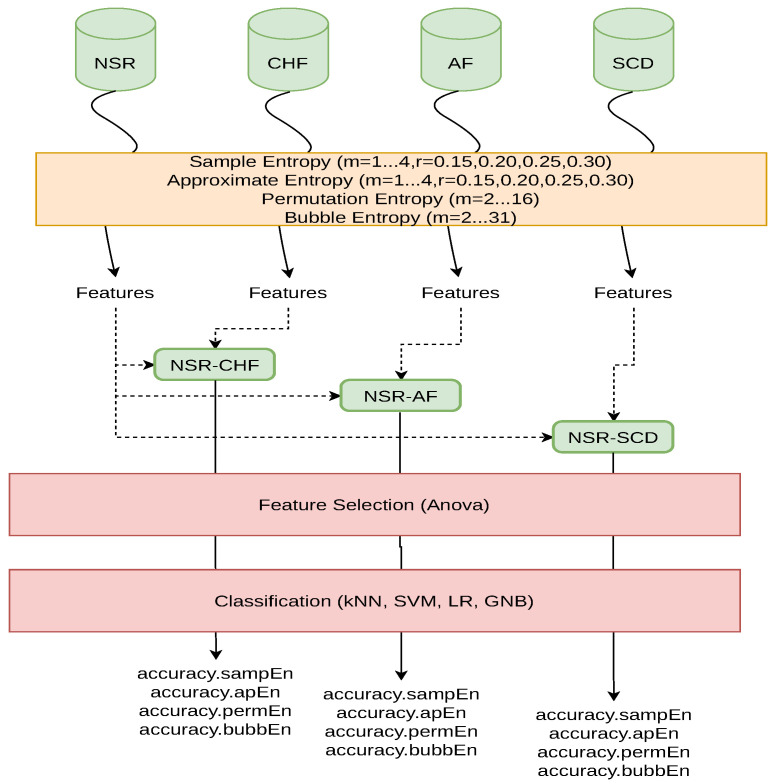
The two-class challenges: for each dataset, features are extracted; three challenges are created, feature selection is applied and classification accuracy is computed.

The two-class challenges are shown in Figure 1. Normal sinus rhythm (NSR), congestive heart failure (CHF), atrial fibrillation (AF), and sudden cardiac death (SCD) recordings were analyzed and features were extracted using all four entropy measures: Sample entropy, Approximate entropy, Permutation entropy and Bubble entropy.

Once the features were extracted, they were paired between datasets to form comparative sets: NSR-CHF, NSR-AF, and NSR-SCD. These paired features were then subjected to a feature selection step to identify the most relevant ones for distinguishing between the conditions. The selected features were subsequently fed into four classification algorithms: k-Nearest Neighbors (kNN), Support Vector Machine (SVM), Logistic Regression (LR), and Gaussian Naive Bayes (GNB).

The classification results were quantified in terms of accuracy and F1-score, for each type of entropy measure separately and for each dataset pair. Solid arrows in the diagram represent the primary flow of data and features, while dashed arrows indicate the pairing of features between datasets.

**Figure 2:**
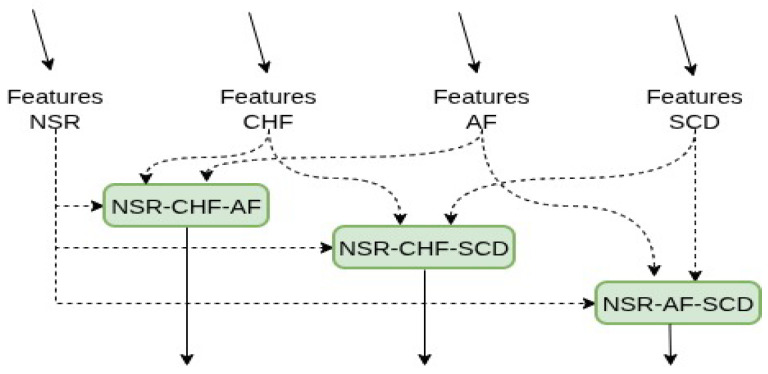
The three-class challenges, one for each possible combination containing NSR.

The three-class classification challenges gave us the opportunity to test the methods with a more difficult classification. The patients’ datasets were combined into three groups of two, and all possible combinations were included. In each of these three groups, the NSR dataset was added, forming three groups of three datasets each. In this way, three three-class classification challenges were created, as shown in Figure 2: NSR-CHF-AF, NSR-CHF-SCD, and NSR-AF-SCD. The rest of the procedure was the same as the two-class challenges.

We performed two different experiments with different signal lengths. In the first experiment, we use the first 100,000 samples of the time series, where each sample corresponds to an RR interval. Since several entropy-based metrics perform more reliably on time series of equal length, we truncated the signals to the minimum mean length across the databases, which is 48,000 (from the AF database) RR intervals.

Approximate entropy is the most typical example. In [28], the size of the signal is recommended to be between $10^{m}$ and $30^{m}$, *m* between 1 and 3 and *r* between 0.1 and 0.25. In our experiments, we included all these suggested values.

Another relevant issue is stationarity. The definition of entropy assumes that the input time series is stationary; however, physiological signals are inherently non-stationary. In this study, entropy measures are employed as practical tools for entropy estimation rather than as strict theoretical quantities. To mitigate the effects of non-stationarity, the signals were filtered to remove artifacts that exacerbate these effects.

RR interval series were filtered using a robust interquartile range (IQR) criterion. For each subject, the first and third quartiles ($Q_{1}$, $Q_{3}$) were computed and $\mathrm{IQR}=Q_{3}-Q_{1}$. Intervals outside

$$[\;Q_{1}-\alpha \cdot \mathrm{IQR},\;Q_{3}+\alpha \cdot \mathrm{IQR}\;],\;\;\;\;\alpha =3$$

were discarded as artifacts. For highly irregular rhythms (e.g., atrial fibrillation and pre-sudden cardiac death recordings), the threshold α was adaptively relaxed to ensure that no more than 3% of RR intervals were removed, thereby preserving pathological variability while eliminating extreme outliers.

### 3.3. Machine Learning Pipeline

We conducted all machine learning classifications with a consistent evaluation framework to enable a fair comparison among the entropy definitions, while preventing information leakage. Model performance was assessed using stratified 10-fold cross-validation, in which nine folds were used for training and the remaining fold for testing; stratification preserved the class distribution and ensured adequate representation of the minority class in each split. Each fold contained complete records/subjects. No data from the same subject appeared in both training and test sets.

A structured pipeline was implemented such that data standardization and feature selection—ranging for each entropy metric from 1 to all computed features—were performed exclusively on the training data within each fold. The same fitted transformations were subsequently applied to the corresponding test fold. This procedure ensured the absence of data leakage and maintained comparability across experiments.

For each configuration, the mean test accuracy across the 10 folds was computed. The entire process was repeated both for all machine learning models and feature subset sizes, and the maximum achieved accuracy along with F1-score were reported.

Hyperparameter tuning is an important aspect of model training that can influence a model’s performance. In order to maintain a controlled and comparable experimental framework, we restricted all models to predefined and limited parameter settings rather than performing extensive optimization.

Four machine learning models were considered. Gaussian Naive Bayes was employed using its default configuration, requiring no explicit hyperparameter selection. For k-Nearest Neighbors, the number of neighbors was evaluated over odd values within the range k∈[3,25] to avoid ties while preserving a bounded search space. Logistic Regression was implemented with L2 regularization, regularization strength C=1, the Limited-memory Broyden–Fletcher–Goldfarb–Shanno (L-BFGS) solver, and class weights adjusted to account for class imbalance, maintaining a fixed configuration across all experiments. Support Vector Classifiers were evaluated using multiple kernel functions (“linear”, “poly”, “rbf”, and “sigmoid”), each treated as a distinct model family with fixed parameters C=1, *γ* = ‘*scale*’, polynomial degree equal to 3 for the “poly” kernel, and again class weights balanced to mitigate the effects of unequal class sizes. Although some kernel configurations, such as “sigmoid”, were expected to yield limited performance, they were included to ensure a thorough and unbiased investigation of the model space. Across all models and parameter configurations, this approach enabled a systematic comparison under uniform complexity constraints while avoiding overfitting and information leakage.

## 4. Evaluation Results

This section consists of two subsections. The first includes results from the two-class and the three-class challenges presented in the tables. The second subsection is on the evaluation of the features and their contribution to a machine-learning-based classification.

### 4.1. Evaluation Based on Accuracy and F1-Score

Table 2 and Table 3 correspond to the NSR-CHF challenge, Table 4 and Table 5 to the NSR-AF and Table 6 and Table 7 to the NSR-SCD. Table 2, Table 4 and Table 6 are for the two-class challenges for time series with size 100,000 samples, while Table 3, Table 5 and Table 7 are for the two-class challenges for time series with size 48,000 samples. They present the accuracy and F1-score achieved by each method separately when using only its own features, and the accuracy and F1-score when all features were combined together. Each line corresponds to a different classifier (GNB, kNN, LR, and SVM). For clarity, we added a row at the end of every table that contains the maximum achieved evaluation metric of every entropy definition.

From the tables, it is easy to conclude that, across all classifiers—except two cases, where Permutation entropy performs better—Bubble entropy consistently yields the highest performance of all entropy measures. Bubble entropy captures class-separating information that the traditional entropy measures do not. This is a strong and consistent trend. Only the “All” column typically exceeds Bubble entropy, since complementary information from the other entropy features adds marginal value when combined. The superiority of Bubble entropy is not classifier-dependent; it reflects the feature’s intrinsic discriminative quality. This strengthens the argument that Bubble entropy captures dynamical or nonlinear characteristics efficiently.

The three-class challenges reinforce the above conclusions. The results are summarized in Table 8, Table 9 and Table 10. The observations are consistent with those obtained from the two-class challenge. Overall, Bubble entropy still yields the strongest classification performance; however, in one case (the NSR–AF–SCD challenge), Permutation entropy achieves higher accuracy and F1-score accordingly.

In conclusion, Bubble entropy tends to outperform the other three entropy definitions in the evaluation tests we performed.

### 4.2. Evaluation Based on Feature Informativeness

The second part of our evaluation focuses on feature informativeness. The extracted features are assessed using widely adopted and well-established feature evaluation metrics. We will give short descriptions of the methods in the following.

One-way ANOVA [29] tests whether a feature’s mean differs across *K* groups. Let $x_{ij}$ be the *j*-th sample in group *i* (i=1,…,K, $j=1,\ldots ,n_{i}$), and $N=\sum_{i=1}^{K}n_{i}$.

$${\bar{x}}_{i}=\frac{1}{n_{i}}\sum_{j=1}^{n_{i}}x_{ij},\;\bar{x}=\frac{1}{N}\sum_{i=1}^{K}\sum_{j=1}^{n_{i}}x_{ij}$$

$$\mathrm{SSB}=\sum_{i=1}^{K}n_{i}{\left({\bar{x}}_{i}-\bar{x}\right)}^{2},\;\mathrm{SSW}=\sum_{i=1}^{K}\sum_{j=1}^{n_{i}}{\left(x_{ij}-{\bar{x}}_{i}\right)}^{2}$$

$$F=\frac{\mathrm{SSB}/(K-1)}{\mathrm{SSW}/(N-K)}$$

A larger *F* indicates stronger discriminative ability of the feature.

Mutual Information (MI) [30] measures the statistical dependency between a feature *X* and the class label *Y*:

$$I\left(X;Y\right)=\sum_{x}\sum_{y}p\left(x,y\right)\log\frac{p(x,y)}{p(x)p(y)}$$

For continuous-valued features, MI is defined analogously using probability density functions and integrals. In this work, MI is estimated using the non-parametric k-Nearest Neighbors method implemented in mutual_info_classif (scikit-learn). Higher I(X;Y) indicates stronger dependency and greater feature relevance.

SHapley Additive exPlanations (SHAP) [31] is a model-agnostic method that attributes a prediction to individual feature contributions based on Shapley values from cooperative game theory. Let *F* be the set of all features and j∈F. For any subset S⊆F∖{j}, let $f_{S}\left(x_{S}\right)$ denote the model evaluated using only features in *S*. The contribution of feature *j* is as follows:

$$\phi_{j}=\sum_{S\subseteq F\setminus \{j\}}\frac{|S|!(|F|-|S|-1)!}{|F|!}\left[f_{S\cup \{j\}}\left(x_{S\cup \{j\}}\right)-f_{S}\left(x_{S}\right)\right]$$

The prediction can be decomposed as follows:

$$f\left(x\right)=\phi_{0}+\sum_{j\in F}\phi_{j}$$

A larger $|\phi_{j}|$ indicates greater influence of feature *j*. Global importance is obtained by averaging $|\phi_{j}|$ over all samples. In this work, these averaged absolute SHAP values are used to rank features, computed using a linear SHAP explainer for Logistic Regression with interventional feature perturbation.

Permutation Importance (PERM) [32] evaluates feature relevance by measuring the decrease in model performance when the feature values are randomly permuted. Let *f* be a trained model and $D={\left\{\left(x^{\left(i\right)},y^{\left(i\right)}\right)\right\}}_{i=1}^{N}$ a dataset. Let M(f,D) denote a performance metric (e.g., accuracy). For feature *j*, let $D^{\pi_{j}}$ be the dataset where the values of feature *j* are randomly permuted across all samples. The importance of feature *j* is defined as follows:

$$I_{j}=M\left(f,D\right)-M\left(f,D^{\pi_{j}}\right)$$

A larger $I_{j}$ indicates that permuting feature *j* leads to a greater drop in performance, implying higher importance. In this work, $I_{j}$ is estimated using repeated random permutations (n_repeats=50), and the mean decrease in accuracy is used to rank features.

**Figure 3:**
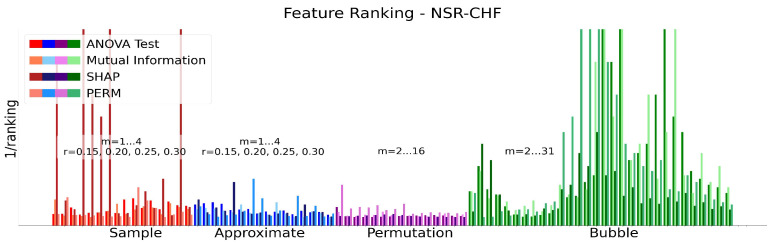
Feature ranking for the NSR–CHF challenge, using up to 100,000 samples. Higher bars correspond to more informative features. The features with the highest importance are produced by Bubble entropy.

**Figure 4:**
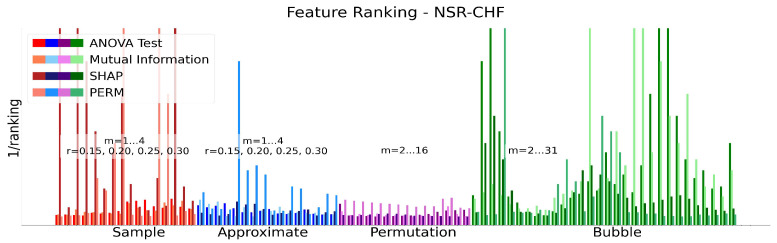
Feature ranking for the NSR–CHF challenge, using up to 48,000 samples. Higher bars correspond to more informative features. The features with the highest importance are produced by Bubble entropy.

**Figure 5:**
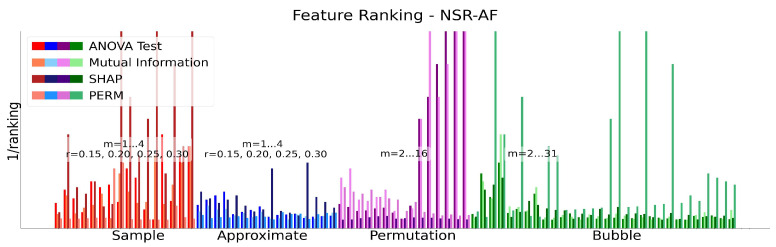
Feature ranking for the NSR–AF challenge, using up to 100,000 samples. Higher bars correspond to more informative features. The features with the highest importance are produced by Sample entropy and Permutation entropy.

**Figure 6:**
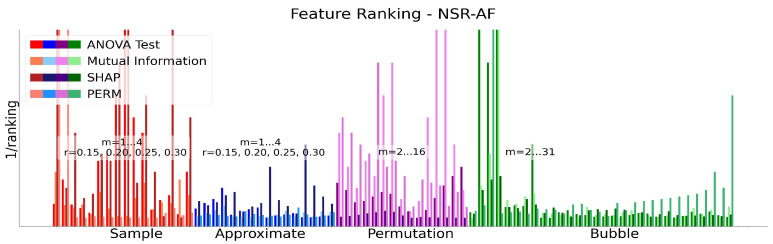
Feature ranking for the NSR–AF challenge, using up to 48,000 samples. Higher bars correspond to more informative features. The features with the highest importance are produced by Sample entropy and Permutation entropy.

**Figure 7:**
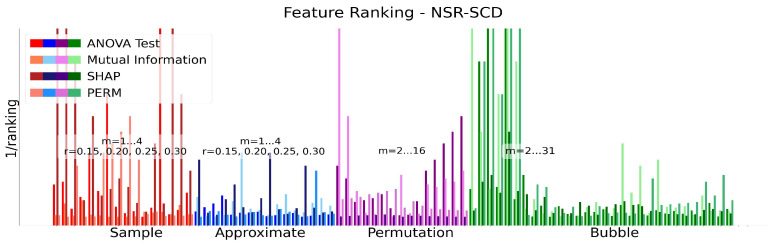
Feature ranking for the NSR–SCD challenge, using up to 100,000 samples. Higher bars correspond to more informative features. The features with the highest importance are produced by Bubble entropy.

**Figure 8:**
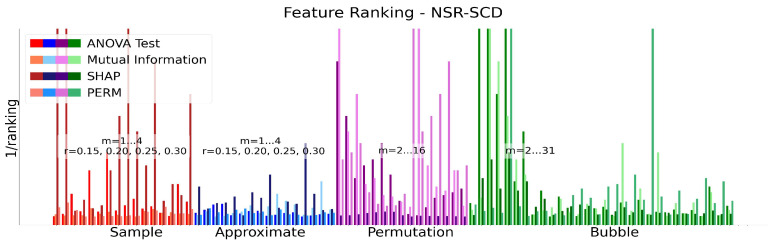
Feature ranking for the NSR–SCD challenge, using up to 48,000 samples. Higher bars correspond to more informative features. The features with the highest importance are produced by Bubble entropy.

Features are all grouped together and ranked according to the aforementioned measures. The inverse of the rank (1/rank) is then plotted, grouped by entropy method. Figure 3, Figure 4, Figure 5, Figure 6, Figure 7 and Figure 8 summarizes the results. Figure 3, Figure 5 and Figure 7 present the results for a signal length of 100,000 samples and Figure 4, Figure 6 and Figure 8 for 48,000. Larger bar values correspond to more important features, whereas smaller values indicate less informative ones. From the figures, it is evident that the Bubble entropy features are consistently identified as the most informative in the NSR-CHF challenge, which is in agreement with the accuracy and F1-scores reported in Section 4.1. However, Permutation entropy and Sample entropy manage to compete with Bubble entropy in both the NSR-AF and NSR-SCD challenges.

**Figure 9:**
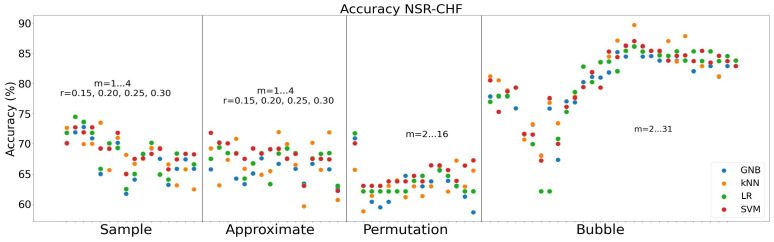
Classification with only one feature (NSR-CHF challenge). Bubble entropy achieves the best classification results.

A final evaluation criterion was the ability of a feature to discriminate between the two classes independently of other features. To assess this, we provided each of the four classifiers with a single feature as input and recorded the resulting accuracy. The results were then plotted in graphs, grouped according to the entropy measure. Here, we show one representative plot from the NSR-CHF challenge (Figure 9). The plot again demonstrates that Bubble entropy generated features yielding the highest accuracies. Similar patterns were observed across all other challenges.

In conclusion, Bubble entropy presented the most informative features.

## 5. Discussion

Bubble entropy was originally proposed as an entropy definition that frees the user of the need to estimate parameters. Owing to its dependence only on the size of the embedding space and its very low computational complexity, the method can combine a large number of features from different embedding dimensions. Consequently, all reasonable features can be included, relieving the programmer of the need to select among the best. In contrast, Sample entropy and Approximate entropy are typically limited to values up to m=4. For any value of *r* and *m*, in a stationary process, Sample entropy and Approximate entropy converge, when the number of points is sufficient, to the same exact value. They do not estimate different effects at different scales at all.

Bubble entropy has been presented in detail and extensively discussed in [1,2,3]. However, it still needs evaluation in diverse scientific fields. In this paper, we assess Bubble entropy as a discriminative tool for RR interval sequences. Data from healthy subjects were used as a reference group, whereas pathological conditions were represented by recordings from individuals with congestive heart failure, atrial fibrillation, and sudden cardiac death. A set of features derived from the well-established definitions of Sample entropy, Approximate entropy, and Permutation entropy was extracted, along with those from Bubble entropy, and evaluated. Multiple classifiers were employed to compute the discriminative performance, with assessment based on classification accuracy and the selected feature subsets. The comparative analysis indicates that Bubble entropy consistently achieves superior performance, highlighting its potential.

## 6. Conclusions and Future Work

The purpose of this paper was to evaluate Bubble entropy with RR time series. Publicly available datasets were used for this purpose. Conclusions can be summarized as follows:

- Of the features generated using Bubble entropy, Permutation entropy, Sample entropy, and Approximate entropy, Bubble entropy yielded the most informative features based on four common feature evaluation methods.
- Feeding all features from each method to four machine learning classifiers, Bubble entropy achieved the best accuracies, in six out of nine classification challenges. In the other three challenges, Permutation entropy gave the highest accuracy.
- Using each produced feature separately as an input to a classifier, features from Bubble entropy presented the best classifications.

We can conclude that Bubble entropy outperformed in this evaluation the three most widely used and widely accepted entropy definitions.

We plan to extend the evaluation of Bubble entropy to other physiological signals or signals from diverse scientific fields, beyond biomedical engineering. We also plan to include in the evaluation recent entropy definitions, which are also very promising [23].

## Figures and Tables

**Table 1:** Key statistical properties.

Database	Mean Value	SD	Mean Length	Min. Length
NSRDB	0.772	0.173	100,252.22	81,641
NSR2DB	0.781	0.164	107,405.90	76,810
CHFDB	0.680	0.135	107,431.80	75,415
CHF2DB	0.686	0.124	113,414.06	90,749
AF	0.760	0.195	48,039.08	32,035
SCD	0.855	0.275	80,953.82	17,087

**Table 2:** Classification scores for the NSR-CHF dataset, using up to 100,000 samples.

	Accuracy (%)					Weighted F1-Score (%)				
Classifiers	SampEn	ApEn	PermEn	BubbEn	All	SampEn	ApEn	PermEn	BubbEn	All
GNB	67.05	74.17	69.85	91.52	89.70	64.41	73.34	64.93	90.19	90.27
kNN	73.26	76.97	72.58	88.03	90.45	70.57	73.57	69.83	87.00	90.00
LR	70.68	81.89	69.70	87.95	89.70	70.49	77.84	70.29	85.71	89.70
SVM	74.92	82.73	70.76	89.77	90.45	74.58	79.65	68.36	87.47	91.23
**Max**	74.92	82.73	72.58	**91.52**	90.45	74.58	79.65	70.29	**90.19**	91.23

Bold numbers indicate the best classification score.

**Table 3:** Classification scores for the NSR-CHF dataset, using up to 48,000 samples.

	Accuracy (%)					Weighted F1-Score (%)				
Classifiers	SampEn	ApEn	PermEn	BubbEn	All	SampEn	ApEn	PermEn	BubbEn	All
GNB	68.18	68.18	68.26	86.29	86.36	61.57	66.83	62.82	85.79	86.28
kNN	72.73	75.91	69.77	86.44	87.20	64.55	73.70	64.83	83.62	87.36
LR	74.17	81.89	66.14	83.64	88.03	70.40	81.19	65.50	80.73	85.04
SVM	76.67	82.73	69.70	87.20	89.77	73.73	82.73	67.06	83.87	90.04
**Max**	76.67	82.73	69.77	**87.20**	89.77	73.73	82.73	67.06	**85.79**	90.04

Bold numbers indicate the best classification score.

**Table 4:** Classification scores for the NSR-AF dataset, using up to 100,000 samples.

	Accuracy (%)					Weighted F1-Score (%)				
Classifiers	SampEn	ApEn	PermEn	BubbEn	All	SampEn	ApEn	PermEn	BubbEn	All
GNB	87.78	89.89	99.00	94.89	100	84.65	88.41	99.03	94.38	100
kNN	87.78	94.00	100.00	95.89	100	85.85	92.50	100.00	95.42	100
LR	88.78	93.78	100.00	91.89	100	85.42	93.90	100.00	92.26	100
SVM	88.67	95.89	100.00	94.89	100	87.34	95.04	100.00	95.42	100
**Max**	88.78	95.89	**100.00**	95.89	100	87.34	95.04	**100.00**	95.42	100

Bold numbers indicate the best classification score.

**Table 5:** Classification scores for the NSR-AF dataset, using up to 48,000 samples.

	Accuracy (%)					Weighted F1-Score (%)				
Classifiers	SampEn	ApEn	PermEn	BubbEn	All	SampEn	ApEn	PermEn	BubbEn	All
GNB	85.67	85.67	94.89	92.89	98.00	83.93	83.68	92.38	92.68	99.03
kNN	86.78	91.67	94.89	94.89	98.00	84.88	89.91	93.97	94.49	97.67
LR	82.67	94.89	94.78	92.89	98.00	83.03	94.83	93.51	91.41	99.03
SVM	87.67	93.89	95.89	94.89	99.00	84.31	94.17	95.28	94.49	98.93
**Max**	87.67	94.89	**95.89**	94.89	99.00	84.88	94.83	**95.28**	94.49	99.03

Bold numbers indicate the best classification score.

**Table 6:** Classification scores for the NSR-SCD dataset, using up to 100,000 samples.

	Accuracy (%)					Weighted F1-Score (%)				
Classifiers	SampEn	ApEn	PermEn	BubbEn	All	SampEn	ApEn	PermEn	BubbEn	All
GNB	82.00	84.44	86.56	93.67	92.44	76.82	83.80	84.34	92.08	91.90
kNN	88.56	87.56	88.67	92.56	93.56	87.36	85.79	88.06	91.71	90.98
LR	82.22	86.67	83.67	89.56	93.44	81.65	87.58	82.49	89.15	90.64
SVM	82.11	87.78	87.33	92.78	95.56	82.61	89.22	86.02	93.13	95.48
**Max**	88.56	87.78	88.67	**93.67**	95.56	87.36	89.22	88.06	**93.13**	95.48

Bold numbers indicate the best classification score.

**Table 7:** Classification scores for the NSR-SCD dataset, using up to 48,000 samples.

	Accuracy (%)					Weighted F1-Score (%)				
Classifiers	SampEn	ApEn	PermEn	BubbEn	All	SampEn	ApEn	PermEn	BubbEn	All
GNB	79.11	82.89	86.33	91.67	91.56	76.17	77.88	84.58	89.87	90.65
kNN	86.44	87.22	90.67	92.56	92.67	81.28	83.15	89.38	91.52	90.38
LR	80.11	83.89	85.11	91.67	91.56	81.64	84.12	84.87	90.60	92.75
SVM	83.22	88.33	89.44	91.56	93.67	78.89	87.10	88.29	91.64	95.16
**Max**	86.44	88.33	90.67	**92.56**	93.67	81.64	87.10	89.38	**91.64**	95.16

Bold numbers indicate the best classification score.

**Table 8:** Classification scores for the NSR-CHF-AF dataset, using up to 100,000 samples.

	Accuracy (%)					Weighted F1-Score (%)				
Classifiers	SampEn	ApEn	PermEn	BubbEn	All	SampEn	ApEn	PermEn	BubbEn	All
GNB	58.14	63.95	71.67	83.81	85.76	53.46	60.10	66.33	81.33	86.07
kNN	67.33	74.43	78.67	80.90	91.48	62.53	71.30	73.14	80.62	90.31
LR	67.43	75.24	73.10	79.62	90.76	68.01	76.55	71.87	79.52	93.46
SVM	68.14	77.33	78.00	83.14	91.43	66.63	77.25	74.35	83.21	92.75
**Max**	68.14	77.33	78.67	**83.81**	91.48	68.01	77.25	74.35	**83.21**	93.46

Bold numbers indicate the best classification score.

**Table 9:** Classification scores for the NSR-CHF-SCD dataset, using up to 100,000 samples.

	Accuracy (%)					Weighted F1-Score (%)				
Classifiers	SampEn	ApEn	PermEn	BubbEn	All	SampEn	ApEn	PermEn	BubbEn	All
GNB	55.33	61.15	62.58	81.21	82.75	51.77	55.57	56.64	79.23	81.51
kNN	66.92	66.32	67.69	75.49	79.12	60.39	65.76	64.51	73.95	75.90
LR	56.81	69.07	61.10	72.75	81.32	58.08	64.26	60.26	73.58	80.47
SVM	62.69	71.26	66.87	79.84	86.26	60.78	69.13	65.85	77.02	84.66
**Max**	66.92	71.26	67.69	**81.21**	86.26	60.78	69.13	65.85	**79.23**	84.66

Bold numbers indicate the best classification score.

**Table 10:** Classification scores for the NSR-AF-SCD dataset, using up to 100,000 samples.

	Accuracy (%)					Weighted F1-Score (%)				
Classifiers	SampEn	ApEn	PermEn	BubbEn	All	SampEn	ApEn	PermEn	BubbEn	All
GNB	70.00	70.00	85.83	78.33	86.67	61.69	69.51	81.85	77.47	83.45
kNN	75.83	79.17	85.00	79.17	88.33	68.02	73.59	84.48	77.80	85.57
LR	68.33	73.33	79.17	74.17	85.00	67.98	74.04	78.65	73.21	85.16
SVM	70.83	73.33	83.33	82.50	89.17	68.65	72.53	82.85	82.33	87.91
**Max**	75.83	79.17	**85.83**	82.50	89.17	68.65	74.04	**84.48**	82.33	87.91

Bold numbers indicate the best classification score.

## Data Availability

Data used in this work are publicly available on Physionet. The necessasry references have been given in the manuscript.
